# Influenza D Virus Circulation Among Bovines, Swine, Equines, and Wild Boars in Italy: A Sero-Epidemiological Study

**DOI:** 10.3390/pathogens14090891

**Published:** 2025-09-05

**Authors:** Alessandro Falsini, Chiara Coppola, Aurora Fiori, Domenico Buonavoglia, Serena Marchi, Emanuele Montomoli, Francesco Pellegrini, Gianvito Lanave, Vito Martella, Michele Camero, Claudia Maria Trombetta

**Affiliations:** 1 Department of Molecular and Developmental Medicine, University of Siena, 53100 Siena, Italy; alessandro.falsini2@unisi.it (A.F.); chiara.coppola2@unisi.it (C.C.); aurora.fiori@unisi.it (A.F.); serena.marchi2@unisi.it (S.M.); montomoli@unisi.it (E.M.); 2 Department of Veterinary Medicine, University of Bari, 70010 Valenzano, Bari, Italy; domenico.buonavoglia@uniba.it (D.B.); francesco.pellegrini@uniba.it (F.P.); gianvito.lanave@uniba.it (G.L.); vito.martella@uniba.it (V.M.); michele.camero@uniba.it (M.C.); 3 VisMederi srl, 53100 Siena, Italy; 4 Department of Pharmacology and Toxicology, University of Veterinary Medicine, 1078 Budapest, Hungary

**Keywords:** influenza D virus, epidemiology, swine, equine, bovine, wild boar

## Abstract

Influenza D virus (IDV), belonging to the *Orthomyxoviridae* family, was first discovered in 2011 in pigs. Surveys in humans and animals have been carried out to decipher IDV ecology. In this seroepidemiological study, we investigated the circulation of IDV lineages across Italy in livestock and wildlife animals. A total of 1038 animal serum samples (from 246 bovines, 249 swine, 98 equines, and 445 wild boars) were tested using hemagglutination inhibition and virus neutralization assays. The results confirm bovines as the primary reservoir for IDV, with high seroprevalence for both D/660 (87%) and D/OK (80%) strains. Swine and equines demonstrated limited exposure, suggesting they are infrequent spillover hosts. Notably, wild boars showed high seroprevalence, especially for the D/660 lineage (80%), indicating their potential role in a wildlife transmission cycle. Continuous surveillance in both livestock and wildlife is essential to monitor the spread and evolution of IDV.

## 1. Introduction

Influenza D virus (IDV) is a relatively recent member to the *Orthomyxoviridae* family, first isolated in 2011 in swine with influenza-like symptoms in Oklahoma, USA [1]. Although IDV shares structural and genetic similarities with Influenza C virus (ICV), it is classified as a distinct type due to significant antigenic and genetic divergence [2]. IDV has been detected in a wide range of hosts, including cattle, swine, camelids, small ruminants and, more recently, in dogs [3,4,5,6,7,8]. Serological evidence suggests that bovines may serve as the primary reservoir for the virus [3,4,5]. In addition, antibodies against IDV have been detected in humans [6,7,8].

IDV possesses a single-stranded, negative-sense RNA genome segmented into seven parts, encoding at least nine proteins, including the hemagglutinin-esterase fusion (HEF) glycoprotein, which plays a pivotal role in viral attachment and entry [1,9]. Unlike influenza A and B viruses, IDV exhibits a broader host range and a more conserved HEF gene, suggesting lower antigenic drift and posing potential challenges for vaccine design [10]. Experimental infection studies have shown that IDV replicates efficiently in both the upper and lower respiratory tract of cattle, leading to mild to moderate respiratory symptoms. IDV may also predispose animals to secondary bacterial infections within the bovine respiratory disease complex [2,3].

IDV attaches to host cells through its HEF glycoprotein, which specifically binds to 9-O-acetylated sialic acids as cellular receptors. These receptors are abundantly expressed in the respiratory epithelium of cattle and various other mammalian species, correlating with the broad host range observed for IDV [11].

Furthermore, surveillance studies conducted across various geographic regions, including North America, Europe, Asia, and Africa, have identified multiple co-circulating genetic lineages of IDV, such as D/660, D/OK, and D/Japan. Phylogenetic analyses indicate occasional reassortment events and limited geographic compartmentalization among these lineages [4,12]. Overall, IDV exhibits relatively high environmental stability and efficient transmission among cattle, suggesting a potential for widespread dissemination within livestock populations [13].

The zoonotic potential of IDV remains under investigation. However, higher seroprevalence rates among workers with occupational exposure to livestock raise concerns about interspecies transmission and potential public health implications [8]. Studies using both standard human lung epithelial cell lines and primary well-differentiated human airway epithelial cultures have shown that IDV can efficiently infect human respiratory epithelial cells, replicating to high titers comparable to those observed with influenza A virus [14].

Understanding the epidemiology, pathogenicity, and molecular characteristics of IDV is therefore essential for developing effective surveillance strategies and evaluating its potential impact on both animal and human health. In this study, we investigated the prevalence of antibodies against IDV D/660 and D/OK lineages in serum samples from cattle, horses, swine, and wild boars, adopting a multi-species surveillance approach.

## 2. Materials and Methods

### 2.1. Animal Samples

**Figure 1:**
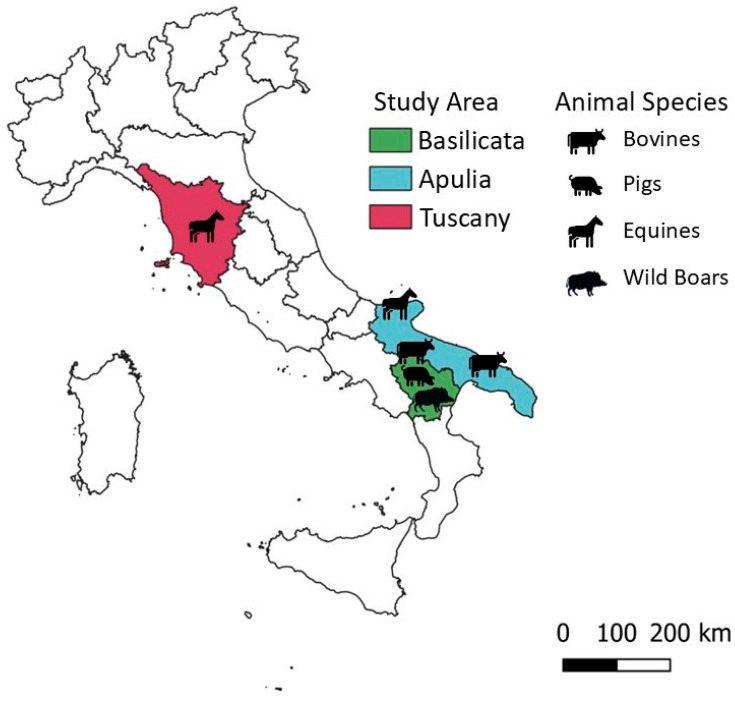
Study area. Map showing the region where animal serum samples were collected. The map was created using QGIS 3.42.3 software to ensure accurate scaling and further modified with Microsoft Power Point.

Animal serum samples were collected from various species over a broad timespan. Specifically, as represented in Figure 1 and resumed in Table 1, 246 bovine serum samples were collected between 2014 and 2015 in Apulia and Basilicata regions, Southern Italy; 249 swine serum samples were collected in 2021 in Apulia region; a total of 98 equine serum samples were collected, 36 in 2001 in Tuscany region (Central Italy) and 62 in 2023 in Apulia; 445 wild boar serum samples were collected in Basilicata region, Southern Italy, during 2020 and 2021.

Bovine serum samples were collected from four different farms, of which three were located in Apulia and one in Basilicata. The maximum distance between the farms was 90 km, with an average distance of approximately 30 km between each other.

Animal blood samples were collected following standard procedures in tubes without anticoagulants. The samples were then centrifuged, and the serum was separated, aliquoted, and stored at −80 °C until use.

All samples were tested in duplicate using the hemagglutination inhibition (HI) and virus neutralization (VN) assays. Wild boar samples were tested only by HI, as the remaining volume was insufficient for additional analyses.

### 2.2. Influenza Viruses

Influenza D/bovine/Oklahoma/660/2013 virus (D/660 lineage, here referred to as D/660), kindly provided by Prof. Feng Li, University of Kentucky, and influenza D/swine/Italy/199724-3/2015 virus (D/OK lineage, here referred to as D/OK), obtained from the European Virus Archive (EVAg), were propagated in Madin-Darby Canine Kidney (MDCK) cells, as previously described [6].

MDCK cell cultures were grown at 37 °C and 5% CO_2_ in Eagle’s Minimum Essential Medium (EMEM) (Euroclone, Pero, MI, Italy) in a humidified incubator, supplemented with 10% *v*/*v* fetal bovine serum (Euroclone, Pero, MI, Italy), 1% *v*/*v* L-glutamine (Euroclone, Pero, MI, Italy), 1% *v*/*v* MEM Non-Essential Amino Acids Solution 100X (Euroclone, Pero, MI, Italy) and 1% *v*/*v* penicillin–streptomycin (Euroclone, Pero, MI, Italy). MDCK cells were seeded in a T175 cm^2^ culture flask at a density of 1 × 10^6^ cells/mL with UltraMDCK supplemented with 1% *v*/*v* penicillin-streptomycin. After 18–20 h, the cell monolayer was washed twice with sterile Dulbecco’s Phosphate Buffer Saline (DPBS), and cells were infected with UltraMDCK containing the respective virus at a Multiplicity of Infection of 0.001. After 1 h of incubation at 37 °C in a humidified atmosphere with 5% CO_2_, 50 mL of UltraMDCK containing a final concentration of 2 mcg/mL of trypsin acetylated (Sigma-Aldrich, Saint Louis, MO, USA) was added to the flask. The infected cells were incubated at 37 °C in a humidified atmosphere with 5% CO_2_ for 72–96 h. The cytopathic effect (CPE) was monitored every day, along with the hemagglutination (HA) titer of the supernatant. At 90% of the CPE, the culture medium was harvested, centrifuged at +4 °C to remove the cell debris, and stored at −80 °C.

### 2.3. Haemagglutination Inhibition Assay

The HI assay was performed as previously reported [6]. All serum samples, including positive and negative controls, were pre-treated with receptor-destroying enzyme (RDE) from Vibrio cholerae (ratio 1:4) (Denka, Tokyo, Japan) for 18 h at 37 °C in a water bath and then heat inactivation for 1 h at 56 °C. Samples were then re-treated with 15% turkey red blood cells (RBCs) (ratio 1:1, final sample dilution 1:10) and tested in duplicate. The HA titer of each IDV strain was determined using 0.35% turkey RBCs and subsequently diluted to 4 HA units per 25 µL. The HA titer was confirmed by back titration, and dilutions were adjusted as necessary. The antibody titer was expressed as the reciprocal of the highest serum dilution showing complete inhibition of agglutination. As the starting dilution was 1:10, titers below the detectable threshold were conventionally expressed as 5 for calculation purposes (half the lowest detection threshold). Serum samples with an average antibody titer of 10 or higher were classified as positive for HI and VN assays [15].

### 2.4. Virus Neutralization Assay

The VN assay was conducted as previously reported [6]. MDCK cells were cultured at 37 °C in a 5% CO_2_ atmosphere and pre-incubated in 96-well plates for 4 h. Serum samples, along with positive and negative controls, were heat-inactivated at 56 °C for 30 min, then twofold serial diluted in UltraMDCK medium (Lonza, Walkersville, MD, USA). Each sample was mixed with an equal volume of virus suspension containing 100 TCID_50_ per well and incubated for 1 h at 37 °C with 5% CO_2_. Subsequently, 100 µL of the virus–serum mixture was transferred to the pre-seeded MDCK cell cultures. Plates were incubated for four days under the same conditions and read for HA activity in the supernatant.

### 2.5. Data Analysis

Data analysis was performed using GraphPad Prism Version 8.4.2 (GraphPad Software) and Microsoft Excel 2019. Data were log transformed. Log normality of distribution was evaluated by the Shapiro–Wilk test. Median, 25th, and 75th percentile was assessed. The non-parametric Kruskal–Wallis test was performed to evaluate statistical significance in antibody titer distributions between the two IDV lineages (D/660 and D/OK) across the different animal species. Statistical significance was set at * *p* < 0.05.

## 3. Results

To investigate the prevalence of IDV D/660 and D/OK strains in different animal species in Italy, we tested serum samples from multiple hosts collected over different years (2001, 2014, 2015, 2021, and 2023) and Italian regions (Tuscany, Apulia, and Basilicata)

As shown in Table 2, cows showed mainly positive titers (≥10) to both viral strains, with 87% of samples being positive for D/660 and 80% for D/OK. The proportion of samples with non-detectable titers (≤5) remained as low as 14% and 20% for D/660 and D/OK, respectively. These data strongly support the role of cattle as the primary reservoir for IDV, according to the current epidemiological evidence and literature [3].

Pigs showed a different serological profile, with the majority of samples showing low or undetectable titers (≤5:69% for D/660 and 71% for D/OK). Only a small proportion exhibited higher titers, with an overall seroprevalence of 31% for D/660 and 29% for D/OK. This pattern suggests limited exposure to IDV in swine populations and supports the notion that this animal species is unlikely to serve as significant reservoirs for the virus under current conditions.

Horses exhibited an almost complete lack of serological response, with HI titers ≤ 5 detected in 95–99% of samples. Positive titers (≥10) were observed in only 5% of samples for D/660 and 1% for D/OK. Notably, among the five IDV-positive horses, one animal sampled in 2001 showed a HI titer of 10 for both IDV lineages, D/660 and D/OK, although the first reported isolation of IDV dates back to 2011 [1].

Of note, wild boars exhibited an intermediate serological profile compared to the other animal species investigated in this study. For the D/660 strain, 57% of samples showed high titers (≥40), with an overall seroprevalence (≥10) of 80%. In contrast, for the D/OK, the percentage of high titers dropped to 36%, and overall seroprevalence (≥10) decreased to 56%, accompanied by an increase in low titers (≤5) from 19% to 44%. Some serum samples were reactive for both IDV lineages; however, in most cases, reactivity was higher to D/660 than to D/OK.

Some samples, mainly from bovines and wild boars, were reactive to both D/660 and D/OK IDV lineages (Table 2). In particular, cattle samples showed positive titers (≥10) to both lineages, with a seroprevalence of 80%, while wild boars showed 51% seroprevalence. In contrast, a lower level of cross-reactivity was observed in swine (20% seroprevalence) and horses (1% seroprevalence).

**Figure 2:**
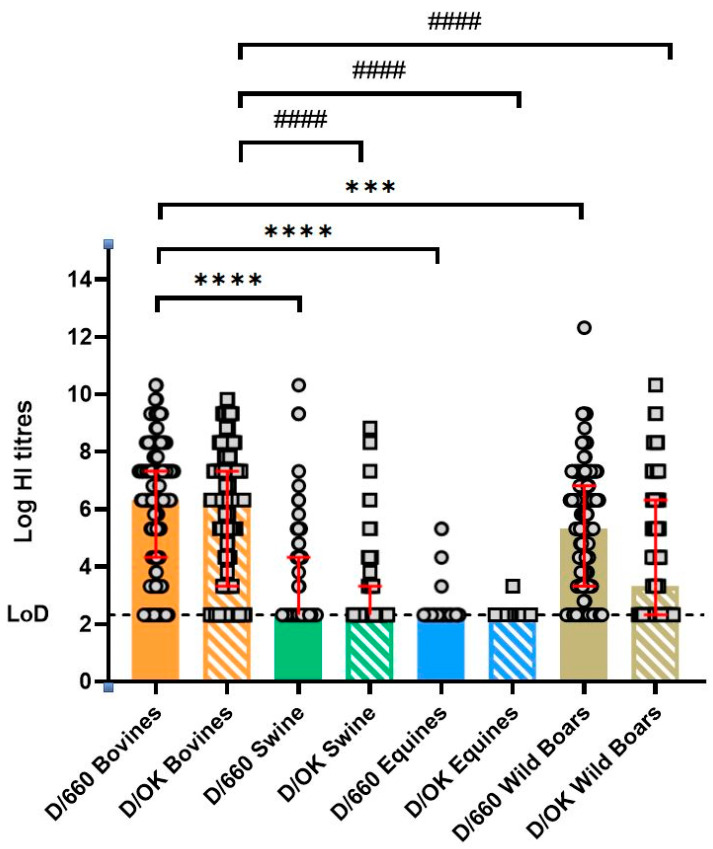
Serum samples from cow, swine, horse, and wild boar were tested for antibodies against IDV D/660 and D/OK. HI titers are shown on a logarithmic scale, with each dot and circle representing an individual animal. Red bars represent median and interquartile range of replicates. The dashed line represents the limit of detection (titer = 10). Statistical significance between groups was determined using Kruskal–Wallis with Dunn’s multiple comparison test. Statistical significance: **** *p* < 0.0001; *** *p* < 0.001; #### *p* < 0.0001 (* refers to D/660, # refers to D/OK).

Figure 2 summarizes the HI results, highlighting the higher prevalence of positive samples in cattle compared to swine, horse, and wild boars. The differences in HI titers for both D/660 and D/OK strains in bovine samples are statistically significant compared to all other animal groups, with *p*-values < 0.0001 or <0.001.

The VN assay largely confirmed the patterns observed in the HI test, with only minor differences, as shown in Table 3.

The higher proportion of low-reacting samples in the VN assay (e.g., in swine) may reflect its higher specificity, suggesting that some HI-positive samples were weakly reactive or non-neutralizing.

## 4. Discussion

In this study, we conducted a seroepidemiological investigation to assess IDV circulation among farmed and wild animals in Italian regions. The HI assay revealed the presence of IDV antibodies across different animal species, reinforcing the notion that cattle may serve as the primary reservoir and revealing variable exposure patterns in wild boars.

A high IDV seropevalence was observed in cattle, with 87% and 80% of samples testing positive for the D/660 and D/OK lineages, respectively. These findings align with numerous reports worldwide, which have documented seropositivity rates up to 80% in bovines [16]. Epidemiological and virological evidence suggests that IDV infection in cattle is associated with the bovine respiratory disease complex [17] along with inflammation in their respiratory tracts, which facilitates viral transmission [3]. Consequently, cows play a crucial role in maintaining IDV transmission cycles and could serve as sentinel species for surveillance programs.

A notable observation arises from the high seroprevalence of IDV in cattle samples collected as early as 2014–2015, despite the virus being first identified only in 2011 in the USA [1]. This may suggest that IDV was already circulating in livestock animals well before its initial detection. Furthermore, several animals tested positive for antibodies against the D/660 lineage, which had not been reported as circulating in Italy during that period. This raises the possibility that a D/660-like virus was introduced earlier than previously documented in Italy, or that exposure to an ancestral IDV strain elicited cross-reactive antibodies against both D/OK and D/660 lineages [18].

On the other hand, swine exhibited low or undetectable HI titers (≤5), with only 13% (D/660) and 8% (D/OK) of animals showing higher titers (HI titers ≥ 40). These findings align with previous studies from North America and Europe, which report similar low seroprevalence rates in domestic swine populations. Indeed, only 19.1% of feral swine in the USA [19] and approximately 5.8% of swine serum samples from Ireland tested positive for IDV [20]. In France, seroprevalence was as low as 1.6% in domestic swine serum samples tested by HI [21]. Taken together, these data support the hypothesis that swine may act as spillover hosts rather than serving as self-sustaining reservoirs of IDV.

Interestingly, wild boars sampled in the same region (Basilicata) and during the same period (2020–2021) exhibited high seroprevalence rates for IDV, up to 80% for D/660 and 56% for D/OK. Notably, higher titers were significantly more frequent against the D/660 lineage than D/OK, supporting previous observations that D/660 has replaced D/OK as the dominant circulating lineage [9]. Studies in wildlife have reported widespread exposure to IDV, including 19.1% seropositivity in feral swine across various regions of the USA [22] and in France [21]. Although a very low IDV prevalence (0.98%) was previously reported in wild ungulates in Italy [23], our findings suggest a potential role for wild boars in sustaining IDV transmission cycle in wildlife. Moreover, seroprevalence analysis in wild boar samples revealed a clustered distribution: while some groups were negative, others were consistently positive. This pattern suggests that IDV circulation may be localized, with certain areas harboring infected wild boar populations. The movement of these different groups in the natural environment could facilitate the spread of IDV, potentially expanding its geographic distribution. Additionally, the risk of virus transmission between wild boars and domestic pigs should be considered higher in areas with semi-intensive and free-range farming systems, such as the Basilicata region [24].

Equine samples exhibited minimal HI reactivity, with titers ≤ 5 in at least 95% of samples, and only 5% animals displaying a detectable HI response. Only two samples showed titers ≥ 40 against D/660 lineage, and none reached this threshold for D/OK. These findings are consistent with previous reports suggesting limited exposure or susceptibility of horses to IDV [22]. However, experimental studies demonstrate that the IDV can replicate in the equine respiratory tract and induce seroconversion [25]. Serological investigations have also confirmed the susceptibility to IDV of equine populations in the Midwestern United States. In a study by Nedland et al. (2018), 364 equine serum samples collected from 141 farms were screened, revealing antibodies against both D/OK (12%) and D/660 (11%) lineages [26]. Interestingly, one of the five equine samples analyzed in the present study, collected in 2001, showed a HI titer of 10 against both D/660 and D/OK despite IDV being first isolated only in 2011 [1]. This sample tested negative for ICV, ruling out potential cross-reactivity between IDV and ICV in the serological assay. The detection of IDV antibodies nearly a decade before its official isolation highlights the need for retrospective studies and deeper investigation into the evolutionary history of the virus. Conversely, the IDV-positive equine samples collected in 2023 were reactive only to the D/660 lineage.

It is noteworthy that some samples tested positive for both IDV lineages. The high proportion of samples reactive to both D/660 and D/OK, particularly in bovines (80%) and wild boars (51%), suggests strong cross-reactive antibody responses. This observation aligns with previous reports [11] indicating partial antigenic overlap between the two lineages and may explain the detection of antibodies against a lineage not known to be circulating in a specific region or time. Although such cross-reactivity complicates lineage-specific prevalence estimates, it also implies that infection with one lineage may confer partial immunity to the other.

An important area of investigation for IDV is the assessment of its zoonotic risks. Human samples collected in the USA and Canada in 2011 showed an IDV seroprevalence of 1.3%, while a study conducted in Italy reported a seroprevalence rates in the general population ranging from 5.1% in 2005 to 46% in 2014 [1,6]. Additionally, a retrospective serological study involving 82 Italian swine veterinarians found evidence of IDV exposure as early as 2004, well before the virus was first identified identification in 2011. Specifically, 4.9% of the samples tested positive for antibodies against the D/660 lineage and 2.4% for the D/OK lineage [7]. Molecular surveillance of respiratory viruses using bioaerosol sampling has also detected IDV in an airport, raising questions about how a livestock-associated virus could contaminate an anthropized environment [27]. Similarly, 27% of aerosol samples collected at Duke University Hospital tested positive for respiratory viruses, including IDV [28]. Despite these findings, there is currently no direct evidence of IDV infection in humans, and no clinical cases have been reported to date. However, the detection of IDV genetic material in nasal swabs from pig farmers [29] suggests potential human susceptibility [22]. Importantly, the presence of viral RNA does not necessarily indicate an active infection or productive viral replication. Viral particles could be transiently present in the upper respiratory tract due to environmental exposure or inhalation of contaminated aerosols, without actual replication in host cells. In such cases, virus isolation, antigen detection, or evidence of seroconversion would be required to confirm active infection. These findings underscore the need for continued investigation into IDV zoonotic risk and highlight a possible occupational exposure hazard. Moreover, the D/660 lineage exhibits a broader receptor-binding tropism than D/OK, particularly in certain wildlife species. While both lineages bind equally to the respiratory tract of cattle, supporting the role of bovines as the primary reservoir, D/660 shows enhanced binding capacity in species where D/OK demonstrates limited attachment, such as the water buffalo and Asian elephant. This suggests that D/660 may possess structural or glycan recognition differences in its HEF protein, allowing it to engage a wider range of 9-O-acetylated sialic acid receptors [26]. Therefore, D/660 could represent a more adaptable IDV lineage with a potentially greater capacity for interspecies transmission.

While this study provides valuable insights into the circulation of IDV among various animal species in Italy, several limitations should be acknowledged. First, the sample size for some species, particularly equines, was relatively small and may not fully represent the broader population. In addition, sampling was restricted to specific geographic areas, and the absence of samples from humans and wild bovine limits the generalizability of the results.

Although HI and VN assays are reliable for antibody detection, the possibility of cross-reactivity between IDV lineages or with other influenza viruses cannot be entirely excluded, potentially affecting the interpretation of lineage-specific seroprevalence. Future research with larger, more geographically diverse samples and combined serological and molecular approaches is needed to overcome these limitations and provide a more comprehensive understanding of IDV epidemiology. Another limitation is the use of a convenience sample collection, which hindered the ability to extract additional contextual or epidemiological insights. Furthermore, sample collections spanned different years and regions. Only the swine and wild boar samples were collected within the same temporal and geographical context.

Overall, these findings provide evidence for the circulation of multiple IDV lineages among different animal species in southern Italy. Notably, IDV prevalence in wild boars was higher than previously reported, while rates in cattle and swine were consistent with the existing literature. These results highlight the need to expand IDV surveillance to specific animal populations, such as wild boars and bovine, through larger, long-term studies. Surveillance efforts should also be extended to other livestock and wildlife species to improve our understanding of IDV ecology and to better assess its potential zoonotic risk.

## Figures and Tables

**Table 1:** All animal species included in this study are listed, along with the number of samples collected, their geographic locations, and the year of collection.

Animal Species	Number of Samples	Area	Year
Bovine	246	Apulia and Basilicata	2014–2015
Swine	249	Basilicata	2021
Equine	98	Tuscany and Apulia	2001 and 2021
Wild Boar	445	Basilicata	2020–2021

**Table 2:** The prevalence of samples was reported as follows: negative (≤5), positive with a titer ≥ 10 but <40, and positive with a titer ≥ 40, each reported with the corresponding 95% confidence interval (CI). The overall prevalence, along with the 95% CI, included all positive samples for the Influenza D/bovine/Oklahoma/660/2013 (D/660) and Influenza D/swine/Italy/199724/2015 (D/OK) strains, as assessed by the HI assay.

Animal Species	HI D/660				HI D/OK				HI D/660–D/OK
	≤5	≥10–<40	≥40	**Overall** **seroprevalence** **≥10**	≤5	≥10–<40	≥40	**Overall** **seroprevalence** **≥10**	**D/660 and D/OK overall** **seroprevalence** **≥10**
**Bovine**	14% (34/246) 95% CI 9.76–18.7%	13% (31/246) 95% CI 8.73–17.4%	74% (181/246) 95% CI 67.60–78.98%	**87%** **(212/246)** 95% CI 81.22–90.24%	20% (48/246) 95% CI 14.75–25.02%	10% (25/246) 95% CI 64.19–75.96%	70% (173/246) 95% CI 64.19–75.96%	**80%** **(198/246)** 95% CI 74.98–85.25%	**80%** **(196/246)** 95% CI 74.10–84.52%
**Swine**	69% (172/249) 95% CI 62.93–74.76%	18% (44/249) 95% CI 13.14–22.99%	13% (33/249) 95% CI 9.30–18.11%	**31%** **(77/249)** 95% CI 25.24–37.07%	71% (178/249) 95% CI 65.44–77.01%	21% (52/249) 95% CI 16.01–26.47%	8% (19/249) 95% CI 4.66–11.66%	**29%** **(71/249)** 95% CI 22.99–34.56%	**20%** **(20/249)** 95% CI 4.98–12.13%
**Equine**	95% (93/98) 95% CI 88.49–98.32%	3% (3/98) 95% CI 0.64–8.68%	2% (2/98) 95% CI 0.25–7.18%	**5%** **(5/98)** 95% CI 1.68–11.51%	99% (97/98) 95% CI 94.45–99.97%	1% (1/98) 95% CI 0.03–5.55%	0% (0/98) 95% CI 0.00–3.69%	**1%** **(1/98)** 95% CI 0.03–5.55	**1%** **(1/98)** 95% CI 0.03–5.55
**Wild Boar**	19% (86/445) 95% CI 15.76–23.31%	23% (102/445) 95% CI 19.09–27.11%	57% (252/445) 95% CI 51.88–61.29%	**80%** **(354/445)** 95% CI 75.50–83.20%	44% (198/445) 95% CI 39.81–49.25%	20% (87/445) 95% CI 15.97–23.55%	36% (160/445) 95% CI 31.49–40.61%	**56%** **(247/445)** 95% CI 50.75–60.19%	**51%** **(225/445)** 95% CI 45.781–55.30%

**Table 3:** The prevalence of samples was reported as follows: negative (≤5), positive with a titer ≥ 10 but <40, and positive with a titer ≥ 40. The overall prevalence included all positive samples for the Influenza D/bovine/Oklahoma/660/2013 (D/660) and Influenza D/swine/Italy/199724/2015 (D/OK) strains, as assessed by the VN assay.

Animal Groups	VN D/660				VN D/OK			
	≤5	≥10–<40	≥40	**Overall** **seroprevalence ≥ 10**	≤5	≥10–<40	≥40	**Overall** **seroprevalence ≥ 10**
**Bovine**	13% (33/246) 95% CI 9.42–18.32%	10% (24/246) 95% CI 6.35–18.32%	77% (189/246) 95% CI 71.05–81.95%	**87%** **(213/246)** 95% CI 81.68–90.58%	29% (43/246) 95% CI 12.95–22.81%	6% (14/246) 95% CI 3.15–9.36%	77% (189/246) 95% CI 71.05–81.95%	**83%** **(203/246)** 95% CI 77.19–87.05%
**Swine**	79% (197/249) 95% CI 73.53–83.99%	11% (28/249) 95% CI 7.60–15.84%	10% (24/249) 95% CI 6.27–14.00%	**21%** **(52/249)** 95% CI 16.01–26.47%	76% (190/249) 95% CI 70.53–81.45%	14% (34/249) 95% CI 9.64–18.55%	10% (25/249) 95% CI 6.60–14.46%	**24%** **(59/249)** 95% CI 18.55–29.47%
**Equine**	98% (96/98) 95% CI 92.82–99.75%	2% (2/98) 95% CI 0.25–7.18%	0% (0/98) 95% CI 0–3.69%	**2%** **(2/98)** 95% CI 0.25–7.18%	98% (96/98) 95% CI 92.82–99.75%	2% (2/98) 95% CI 0.25–7.18%	0% (0/98) 95% CI 0–3.69%	**2%** **(2/98)** 95% CI 0.25–7.18%

## Data Availability

The original contributions presented in the study are included in the article; further inquiries can be directed to the corresponding author.
